# High-Resolution Mass Spectrometry and Chemometrics for the Detailed Characterization of Short Endogenous Peptides in Milk By-Products

**DOI:** 10.3390/molecules26216472

**Published:** 2021-10-27

**Authors:** Carmela Maria Montone, Sara Elsa Aita, Chiara Cavaliere, Andrea Cerrato, Aldo Laganà, Susy Piovesana, Anna Laura Capriotti

**Affiliations:** 1 Department of Chemistry, Sapienza University of Rome, Piazzale Aldo Moro 5, 00185 Rome, Italy; carmelamaria.montone@uniroma1.it (C.M.M.); saraelsa.aita@uniroma1.it (S.E.A.); chiara.cavaliere@uniroma1.it (C.C.); aldo.lagana@uniroma1.it (A.L.); susy.piovesana@uniroma1.it (S.P.); annalaura.capriotti@uniroma1.it (A.L.C.); 2 CNR NANOTEC, Campus Ecotekne, University of Salento, Via Monteroni, 73100 Lecce, Italy

**Keywords:** whey, short-chain peptides, cheese-making industry, suspect screening, peptidomics

## Abstract

The process of cheese-making has long been part of human food culture and nowadays dairy represents a large sector of the food industry. Being the main byproduct of cheese-making, the revalorization of milk whey is nowadays one of the primary goals in alignment with the principles of the circular economy. In the present paper, a deep and detailed investigation of short endogenous peptides in milk and its byproducts (whole whey, skimmed whey, and whey permeate) was carried out by high-resolution mass spectrometry, with a dedicated suspect screening data acquisition and data analysis approach. A total of 79 short peptides was tentatively identified, including several sequences already known for their exerted biological activities. An unsupervised chemometric approach was then employed for highlighting the differences in the short peptide content among the four sets of samples. Whole and skimmed whey showed not merely a higher content of short bioactive peptides compared to whole milk, but also a peculiar composition of peptides that are likely generated during the process of cheese-making. The results clearly demonstrate that whey represents a valuable source of bioactive compounds and that the set-up of processes of revalorization of milk byproducts is a promising path in the obtention of high revenue-generating products from dairy industrial waste.

## 1. Introduction

The earliest evidence for cheese-making can be traced back to the sixth millennium BC, representing a critical development for milk preservation in a transportable and long conservation form [1]. Dairy farms and industries had a significant expansion at the turn of the 20th century thanks to the technological advancements of the second industrial revolution [2]. In 2019, the value of the dairy market was estimated to be about 720 billion US dollars worldwide, and it is projected to grow to 1,032 billion dollars by 2024 (2021 report by Statista), given the continuous growth of the world population and the improvement of living conditions in developing countries. The significant growth in the production of milk-derived products, such as milk powder, butter, and cheese, has also led to a massive release of industrial waste [3]. Every year, it is estimated that 4–11 million tons of dairy waste are released into the environment, causing several dangers to biodiversity due to the depletion of dissolved oxygen inducted by the fat constituents [3,4]. The main byproduct of dairy farming is whey, which is produced following casein coagulation in the cheese-making process. Milk whey contains a broad range of bioactive compounds, such as proteins, lipids, carbohydrates, vitamins, and minerals [5], and has been the object of several studies for its revalorization [6,7]. Whey has long been integrated for human consumption in many products, such as albumin whey cheese (e.g., Italian ricotta) and Norwegian brown cheese. However, nowadays, it is still largely lost [6].

Whey proteins constitute 20% of the total protein content in bovine milk and are mainly constituted of a mixture of β-lactoglobulin, α-lactoglobulin, bovine serum albumin, and immunoglobulins [8]. Several studies have been reported to date that study the effects of whey protein hydrolysates for reducing oxidative stress [9], ameliorating insulin resistance [10], antidiabetic action [11], and cytomodulatory and immunomodulatory activity [12]. The interest in whey hydrolysates has not only been limited to bovine milk, with several studies carried out on goat whey [13], camel whey [14,15], and donkey whey [16]. Moreover, whey-derived peptides have also been obtained through other processes, such as protein and whey cheese fermentation [17]. Despite the broad knowledge of whey peptides derived from enzymolysis or fermentation, the peptides originally present in whey have been largely neglected to date [18]. Being independent of digestive processes, endogenous peptides may exert biological activities in the upper gastrointestinal tract—even before protein digestion. In 2013, Baum et al. identified 248 medium-sized endogenous peptides in whole milk [19]. Similarly, Dallas et al. reported hundreds of endogenous peptides in milk whey permeate [20]. The few papers dealing with the identification of endogenous peptides in milk and derivatives have been mainly focused on medium-sized peptide sequences. However, from a nutritional point of view, short-sized peptides (2–4 a.a.) are of more significant interest. Their short sequences allow them to be effectively absorbed intact from the gastrointestinal, preserving their structure [21,22]. As a result, it is presumable that short peptide sequences will not be subject to in vivo transformation that could affect their biological properties. In the present paper, a comprehensive characterization of short peptide sequences was carried out on milk and its byproducts derived from the cheese-making industrial process (i.e., whole milk, whole whey, skimmed whey, and permeated whey), using a dedicated approach based on high-resolution mass spectrometry (HRMS), suspect screening data processing, and chemometrics. The project had the purpose of unraveling the still scarcely known bioactive constituents of milk byproducts that could open up new paths in the revalorization of industrial dairy waste.

## 2. Results and Discussion

### 2.1. HRMS Data Acquisition and Short Peptide Identification

Despite being particularly interesting due to their enhanced biological properties compared to medium- and long-sized amino acid sequences, short peptides have been largely neglected [23]. The commonly employed analytical platforms that peptidomics has borrowed from bottom-up proteomics, and that include nano-HPLC separation, multi-charged ion acquisition, and database search, are not suitable for short amino acid sequences. These compounds, in fact, are more closely related to metabolites than to longer peptide sequences when it comes to untargeted identification. Their small dimensions, in fact, hinder the generation of multi-charged adducts, and singly charged ions are much more likely to be generated. Moreover, the physicochemical properties of short peptide sequences are more dependent on the nature of the a.a., reflecting in more significant variability of the fragmentation pathways and resulting in the need for manual validation for proper identification [24]. Finally, database search, which associates amino acid sequences to existing peptides present in databases, cannot be employed with satisfying results for very short sequences.

For assessing the short peptide profile of milk byproducts derived from the cheese-making industry, whole milk (WM), whole whey (WW), skimmed whey (SW), and permeated whey (PW) were purified with graphitized carbon black (GCB) [25] and analyzed by HPLC coupled to HRMS using a suspect screening approach. A database of all combinations of the 20 natural a.a. in di-, tri-, and tetrapeptides was obtained by MATLAB, filtered to remove repeating masses, and employed to generate a list of *m/z* to be implemented as an inclusion list in the MS method. The inclusion lists allow for compensating of the limits of data-dependent acquisition (DDA) mode, in which the top n most intense ions in each full-scan are sequentially isolated and fragmented. When complex matrices are analyzed (as in the case of milk and its derivatives), several coeluting compounds could cause mask effects on the compounds of interest (e.g., short peptide sequences). When comprehensive lists of *m/z* are available, suspect screening approaches allow bypassing of the mask effects with the selection of the sole *m/z* present in the inclusion list.

For short peptide annotation, a dedicated data processing approach, which was previously implemented on Compound Discoverer by our research group [24], was employed. MS spectra were therefore extracted and aligned, adducts were evaluated and grouped, and MS/MS spectra were associated with the features. Thanks to the fill gaps tool on Compound Discoverer, compounds with low abundances in one of the sets of samples could still be annotated. Whenever the peaks were absent in one of the sets of samples, the noise level was chosen as the area.

Thanks to the described analytical platform, 79 short peptide sequences were tentatively identified after careful manual validation of the features. In Table 1, the annotated short peptides are reported alongside some details, i.e., retention time, proposed formula, experimental *m/z*, MS accuracy, and main diagnostic product ions. Furthermore, in Table S1, further details are available, including adducts, proposed molecular weight, calculated *m/z*, and GRAVY indices. It is important to highlight that MS/MS spectra obtained with higher collisional dissociation (HCD) do not allow the distinguishing of leucine and isoleucine, which would instead require MS^3^ experiments or other fragmentation techniques [26]. The nomenclature Xle was employed throughout the manuscript and Supplementary Materials for indicating that one of the two isomeric a.a. was present in the peptide sequence.

**Figure 1:**
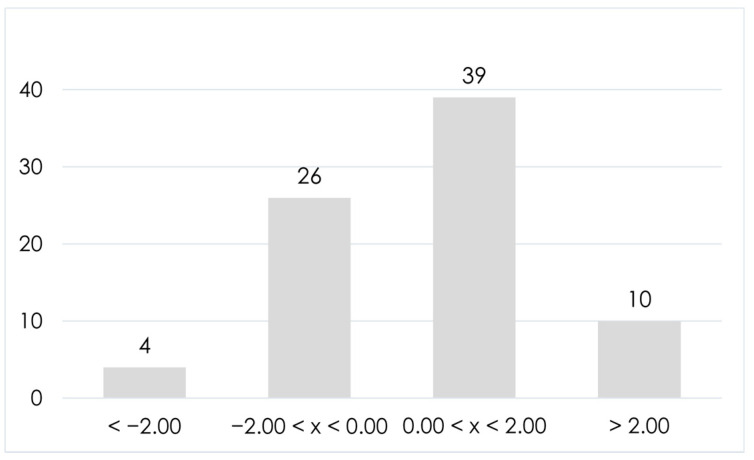
Bar chart reporting the 79 annotated short peptides in milk and milk byproducts based on their GRAVY value.

Of the 79 annotated a.a. sequences, 13 were dipeptides, 31 were tripeptides, and 35 tetrapeptides. The most abundant a.a. were leucine/isoleucine (25.8%), followed by tyrosine (14.3%), phenylalanine (10.8%), arginine (8.5%), and valine (7.7%). On the other hand, aspartic acid and methionine constituted less than 1% of the total a.a. content of the identified short peptides, and no cysteine was reported. The high abundance of branched-chain and aromatic a.a. reflects on the grand average of hydropathicity indexes (GRAVY) of the identified peptides, a parameter that measures the hydrophilicity/hydrophobicity of peptide sequences. The more negative the GRAVY is, the more hydrophilic the sequence is. In general, positive GRAVY values are associated with hydrophobic peptides, whereas negative values are associated with hydrophilic ones. As shown in Figure 1, most annotated short peptides had positive GRAVY values (ca. 62%), with a median GRAVY of 0.45 and ten extremely hydrophobic annotated peptides (GRAVY > 2.00).

The results were dramatically different than those previously reported for milk-derived peptides. In 2014, Dziuba and his coworkers reported 59 antimicrobial peptides from milk proteins with a median GRAVY of −0.4 [27]. Similarly, Piovesana et al. reported that 80% of the total peptides identified from donkey milk protein hydrolysis had a negative GRAVY [28]. Keeping in mind that reversed-phase separation favours hydrophobic peptide separation (and eventually identification), the results could be linked to the origin of such peptides—considering that caseins have a median GRAVY of −0.3, whereas β-lactoglobulin, the main whey protein, is close to 0.

Short peptide sequences are promising bioactive compounds, since, unlike longer peptides, they can be absorbed by the gastrointestinal tract [21]. The a.a. composition of the peptide sequence has a great impact on determining the exerted biological activities. For example, branched-chain and aromatic a.a. (e.g., Ile, Pro, Val, Phe, and Trp), as well as positively charged residues at the *N*-terminus (i.e., His, Lys, and Arg), have been associated with antioxidant activity [29]. The annotated short peptides were searched for in the milk bioactive peptide database (MBPDB) [30], which is dedicated to bioactive peptides in milk and derivatives, and in the BIOPEP-UWM database [31] for their reported bioactivities. Moreover, the sequences were submitted to PeptideRanker, a tool for predicting whether a peptide sequence is bioactive based on a neural network [32]. Forty-nine sequences were either reported in MBPDB or BIOPEP or had a score > 0.50 on PeptideRanker. The results are reported in Table S2.

**Figure 2:**
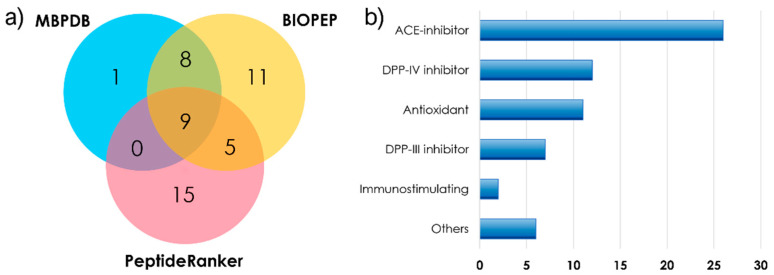
(**a**) Pie-chart reporting the short peptide sequences found in the milk bioactive peptide database (MBPDB) and BIOPEP-UWM, and with a score > 0.50 on PeptideRanker. (**b**) Bar chart reporting the number of peptide sequences found on BIOPEP-UWM per bioactivity.

As shown in Figure 2a, nine peptides were common to the three subsets, i.e., Tyr-Xle, Xle-Trp, Tyr-Pro, Xle-Pro-Tyr, Arg-Tyr-Xle, Xle-Gly-Tyr, Tyr-Xle-Xle, Xle-Arg-Phe-Phe, and Trp-Xle-Gln-Pro. Not unexpectedly, the nine peptides presented several branched-chain and aromatic a.a. (mainly Xle, Tyr, and Trp). Eight more sequences were common to MBPDB and BIOPEP, whereas eleven peptides—mainly dipeptides—were only present in BIOPEP. The fifteen peptides which were predicted by PeptideRanker as possibly bioactive but which were not reported in either MBPDB or BIOPEP demonstrate not merely that short peptides are very likely to exert biological functions, but also that there is still much to discover in the field of short bioactive peptide research.

The reported bioactivities are summarized in Figure 2b based on the results obtained by the BIOPEP-UWM database. Not unexpectedly, most sequences were reported to exert angiotensin-converting enzyme (ACE) inhibitory activity. Peptides can inhibit ACE in three different ways, e.g., the inhibitor way the substrate way, and the prodrug way [33]. Since several short peptides cannot be hydrolyzed by ACE, they can exert a strong inhibitor-like activity, interacting with ACE but preventing the activation of its hydrolyzing activity. Twelve short peptides were reported to inhibit the enzyme dipeptidyl peptidase IV (DPP-IV), which is involved in the increase of blood glucose levels [34]—thus exerting an antidiabetic activity. Other than the eleven peptides with reported antioxidant activity, seven short a.a. sequences were previously linked to inhibitory activity on DPP-III, a metalloprotease that is involved in the cleavage of the N-terminal extremity of various bioactive peptides, including angiotensins and endorphins [35]. Finally, minor reported bioactivities included immuno-stimulating action, α-glucosidase inhibition, anxiolytic activity, renin inhibition, neurological activity, phosphoinositol regulatory activity, and antibacterial action.

### 2.2. Principal Component Analysis of Datasets

For the global evaluation of the short peptide content, an unsupervised chemometric approach was employed, since a proper supervised approach would not have been possible with the available number of samples. The principal component analysis (PCA) on the data matrix obtained following the HRMS data processing was submitted to MetaboAnalyst—a freeware for metabolomics and chemometrics analyses [36]. The 79 annotated short peptide sequences were used as variables. The PCA is the most employed tool for exploratory data analysis, and it is based on the least-square approximation of the data projected on a reduced set of latent variables known as the principal components, which describe the largest possible variability of the experimental datasets [37]. Through the inspection of the PCA results, information on the relationships between the samples is obtained along the principal components on the scores plot, whereas the interpretation of the chemical species of the observed score patterns can be investigated on the loadings plot.

**Figure 3:**
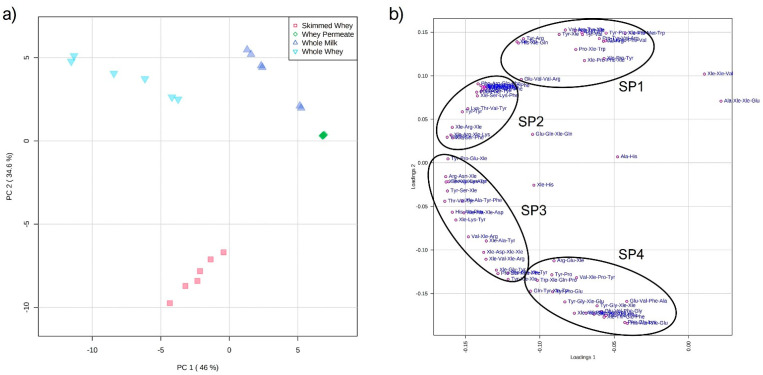
PCA modeling of milk and milk byproduct samples based on the short peptide datasets. (**a**) scores plot; (**b**) loadings plot.

In Figure 3, the PCA modeling of milk and its byproducts is shown. The contribution of the first principal component (PC) was 46.0%, whereas the second PC contributed 34.6% of the total variance (the two PCs combined constituted more than 80% of the total variance). WW and SW samples were clearly discriminated from WM and WP along PC1, with negative values for WW and SW and positive values for WM and WP. On the other hand, WW and SW samples were evidently discriminated along PC2, with positive values for WW (as well as WM and WP) and negative values for SW samples (Figure 3a). The WP samples are hardly differentiated on the scores plot, since their content in most annotated short peptides was negligible compared to the other three sets of samples. The short peptidome profile can be therefore efficiently employed to discriminate WM and the byproducts derived from the cheese-making industrial process.

The loadings plot can be employed for studying the variation in the short peptide profiles of the four sets of samples. In fact, the scores and loadings plot are correlated and the position of each peptide on the loadings plot corresponds to a higher concentration in the samples on the analog position of the scores plot. Four groups of peptides can be highlighted, as shown in Figure 3b, which were labeled Short Peptides 1 (SP1), SP2, SP3, and SP4—with only five remaining peptides that did not fall into any of the four groups. It is worth pointing out that both WW and SW samples showed a generally higher content of short bioactive peptides compared to WM.

**Figure 4:**
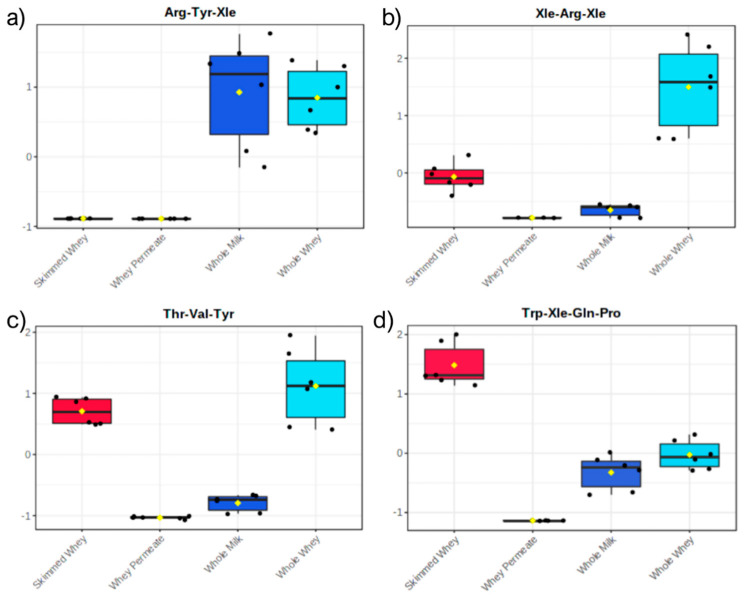
Box and whisker plot of four exemplary short peptides belonging to the four subgroups: (**a**) SP1, characterized by peptides with higher concentrations in whole milk and whole whey, (**b**) SP2, characterized by peptides with higher concentrations in whole whey, (**c**) SP3, characterized by peptides with higher concentrations in whole and skimmed whey, and (**d**) SP4, characterized by peptides with higher concentrations in skimmed whey.

Since only 18 of the annotated peptides were reported in MBPDB, a manual search of the a.a. sequences was manually carried out by inspection of the main milk protein sequences. For this purpose, the four main caseins were considered (α1, α2, β, and κ). Amongst the whey proteins, the a.a. sequences of lactoglobulin, lactalbumin, bovine serum albumin, lactotransferrin, and lactophorin were inspected. The manual sequence search allowed the tentative identification of the origins of 64 of the annotated short peptides—a result that not merely contributed to valorizing the identification platform employed for short peptide annotation, but also demonstrated again the lack of knowledge on short peptides. The protein sources of the short peptides (caseins vs. whey proteins) are reported in Table S2 along with the group to which each compound belonged (SP1 vs. SP2 vs. SP3 vs. SP4 vs. none). In Figure 4, the concentrations of four exemplary peptides from each group in the four sets of samples are reported.

The compounds belonging to the SP1 subset were characterized by higher concentrations in the WM and WW samples (Figure 4a). These 17 peptides were likely derived from endogenous proteases originally present in milk or from protein turnover biocycles in the animals. Compounds belonging to SP1 were relatively shorter compared to the other groups, with many dipeptides that, in light of their extremely short sequence, could not be easily attributed to specific proteins (and could indeed derive from the turnover of other proteins). With regards to the tri- and tetrapeptides, they were mostly matched to the sequences of casein proteins, which hinted at the role of endogenous proteases in their generation.

The second group (SP2) comprised 22 peptides with higher concentrations in WW samples (Figure 4b). Being more concentrated in WW than in WM, these short peptide sequences were likely produced during the process of coagulation of caseins for the separation of the milk solids from the liquid whey. It has been recently reported that short peptides are produced by longer sequences when subject to heating—especially in acid conditions [38]. Similar to peptides belonging to SP1, most of the annotated peptides were matched to casein protein sequences, which could confirm the generation of these peptides during casein precipitation. The lower concentrations of peptides of both SP1 and SP2 groups could be explained by a partial co-precipitation alongside the fat components of milk whey during the skimming process.

With regards to the third group of peptides (SP3, 18 peptides), they were characterized by mostly equally high concentrations in WW and SW (Figure 4c). Similar to SP2, these peptides were likely generated during casein precipitation. Moreover, they could have been derived from the activity of endogenous proteases on whey proteins. As such, the peptides of SP3 were found in both casein and whey protein sequences.

**Figure 5:**
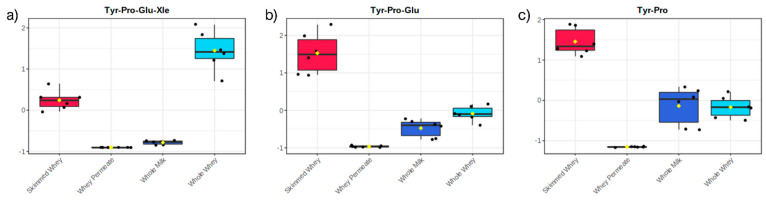
Box and whisker plot showing the abundances in the four sets of samples of Tyr-Pro-Glu-Xle (**a**) and its possible hydrolysis derivatives Tyr-Pro-Glu (**b**) and Tyr-Pro (**c**).

Finally, the peptides belonging to SP4 (17 peptides) were mostly tetrapeptides and were characterized by higher concentrations in SW samples (Figure 4d). Being more abundant in SW than in WW, these sequences were probably produced by the activity of endogenous whey proteases on whey proteins or endogenous medium to long-sized peptides. Similar to the compounds of SP3, these peptides were matched to both casein and whey protein sequences. Evidence for the activity of whey proteases is furnished by some of the annotated short peptides. Tyr-Pro-Glu-Xle, whose sequence was found in that of α-S1 casein (a.a. 161–164), is one of the peptides of the SP3 group and had a somewhat balanced abundance in WW and SW samples, but with higher concentrations in WW (Figure 5a). Among the compounds of SP4, on the other hand, there were two peptides, i.e., Tyr-Pro-Glu and Tyr-Pro, that could be effectively derived from the hydrolysis of Tyr-Pro-Glu-Xle and had, subsequently, higher concentrations in the SW samples (Figure 5b,c).

The findings on the origins of the annotated peptides, which were often derived from casein sequences, disproved the precedent hypothesis on the source of the peptides based on their GRAVY values. The relatively hydrophobic character of the annotated peptides could have derived from the characteristics of the RP separation or from the cleavage preferences of the endogenous proteases.

Compared to WM, WW and SW showed a generally higher content of short endogenous peptides. Moreover, their peptide composition was peculiar, with several compounds present in either WW or SW that were almost absent in WM. On the other hand, WP, which is the final product of the cheese-making industry, showed extremely low abundances of most annotated short peptides—as Figure 4 and Figure 5 suggest.

## 3. Materials and Methods

### 3.1. Chemicals and Materials

Organic solvents of the highest grade available were purchased from VWR International (Milan, Italy). Optima LC–MS grade water and acetonitrile (ACN), used for short peptide analysis, were purchased from Thermo Fisher Scientific (Waltham, MA, USA). Cartridges packed with 500 mg Carbograph 4 were supplied from Lara S.R.L. (Lara S.r.l., Formello, Italy).

### 3.2. Sample Collection

The samples were collected from “Capurso Azienda Casearia srl”, Gioia del Colle (BA, Italy). For this work, the following samples were selected, representing four steps of the cheese-making industry: whole milk (WW), whole whey (WW), skimmed whey (SW), and whey permeate (WP). WW is the byproduct obtained after coagulation of casein proteins through rennet addition for stretched-curd cheese production (cheeses obtained through the method of *pasta filata*). SW is obtained after WW defatting for obtaining whey butter. WP is obtained through reverse osmosis for isolating whey proteins for the obtention of cattle feed. Three distinct samples for each product were furnished by the dairy farm (12 samples) and the samples were freshly aliquoted and stored at −80 °C.

### 3.3. Sample Preparation

For each of the 12 samples, two distinct experiments were carried out (24 data points, 6 per sample type). Concentrated TFA was added to ten milliliters of each sample to reach pH 2. Samples were then centrifuged at 8000× *g* for 35 min at 4 °C to remove any debris. The supernatants were purified on Carbograph 4 cartridges using a previously developed protocol for short peptide purification and pre-concentration [25]. Briefly, the cartridge was first washed to remove impurities, then activated with 10 mL of H_2_O 0.1 mol L^−1^ HCl and conditioned with 10 mL of H_2_O 20 mmol L^−1^ TFA. The extracts were then loaded on the cartridge, which was then washed with 10 mL of H_2_O 20 mmol L^−1^ TFA. The short peptides were eluted with DCM/MeOH 80:20 (*v/v*) with 20 mmol L ^−1^ TFA in backflushing elution mode. The eluates were dried in a thermostated bath at 25 °C under nitrogen flow. The residue was reconstituted in 1 mL of water for subsequent RP separation.

### 3.4. Liquid Chromatography–Mass Spectrometry Analysis

Endogenous short peptide extracts were analyzed by reverse phase chromatography, as described in a previous paper [22]. A Vanquish UHPLC binary pump was used, coupled to a hybrid quadrupole–Orbitrap Q Exactive mass spectrometer (Thermo Fisher Scientific, Bremen, Germany) through a heated electrospray source (HESI). The short peptides were separated by a Kinetex XB-C18 (100 × 2.1 mm, particle size 2.6 μm, Phenomenex, Torrance, CA, USA) operated at 40 °C. Spectra were acquired in the positive ion mode in the range *m/z* 150–750 with a resolution (full width at half maximum, FWHM, *m/z* 200) of 70,000, using a suspect screening approach. An inclusion list containing the exact *m/z* of the protonated ions of all the unique short peptide masses was employed (4980 unique *m/z*). The inclusion lists were prepared using MatLab R2018, as previously described [39]. The acquisition of the higher collisional dissociation (HCD) MS/MS spectra was performed using the top 5 DDA mode at 35% normalized collision energy and 35,000 (FWHM, *m/z* 200) resolution. For each of the 24 data points, three instrumental replicates were run. The average peak areas of the instrumental replicates were employed for statistical analysis.

### 3.5. Short Peptide Identification

The identification of endogenous short peptides was carried out using a dedicated data processing workflow implemented on Compound Discoverer 3.1 (Thermo Fisher Scientific, Bremen, Germany) by our research group, as described in a previous paper [24]. The optimized workflow allowed the extraction of the masses from the RAW data files, according to customized parameters. It also made it possible to predict composition, align the spectra, remove missing blank or MS/MS spectrum signals, and use complete short peptide lists to match the extracted characteristics. The identification of short peptides was confirmed by interpreting the MS/MS spectra aided by mMass, which allows for the in-silico fragmentation of peptides [40].

### 3.6. Statistical Analysis

For statistical analysis, MetaboAnalyst 5.0 [36] was employed. The data matrix obtained following short peptide identification was submitted as a text file that was prepared according to specific indications furnished by the developers. For data filtering, the interquartile range (IQR) was selected, whereas for data scaling, autoscaling (mean-centered and divided by the standard deviation of each variable) was chosen. For the evaluation of the four sets of samples, an unsupervised chemometric approach based on the principal component analysis was chosen.

## 4. Conclusions

Whey represents the main milk byproduct originating from the cheese-making industry, and it is known for being a rich source of valuable bioactive compounds. As demonstrated, both whole and skimmed whey contain a large number of short bioactive peptides (a higher content than whole milk) that are most likely originated following the acid treatment of milk, the heating processes, and the activity of endogenous whey proteases on proteins and longer peptides. The short peptide profile WW and SW, despite the sample being differentiated by the sole skimming process, present several differences that were attributed to the partial co-precipitation of some compounds during fat removal and the activity of endogenous peptidases on whey proteins or long peptide sequences. The short a.a. sequences, being known for their ability to preserve their structure (and subsequently their biological activities) and to be absorbed by the gastrointestinal tissue, short peptide-rich industrial waste could be of significant interest in light of the principles of the circular economy. As such, the renovation of the food industry is intimately linked to the revalorization of byproducts for the generation of high-revenue bioactive compounds.

## Figures and Tables

**Table 1:** Retention times (Rt, min), proposed formulas, experimental *m/z*, accuracy (Δ, ppm), and main diagnostic product ions of the 79 tentatively identified short endogenous peptides in whole milk, whole whey, skimmed whey, and permeate whey.

Name	Rt (min)	Proposed Formula	Exp. *m/z*	Δ (ppm)	Diagnostic Product Ions (*m/z*)
Dipeptides					
Ala-His	0.8	C_9_H_14_N_4_O_3_	227.1138	−0.2	156.0768; 110.0712
Val-Arg	1.9	C_11_H_23_N_5_O_3_	274.1873	−0.1	274.1872; 257.160; 175.1186; 158.0923; 116.0704; 112.0866; 72.0807; 70.0651; 60.0558
Tyr-Gln	2.5	C_14_H_19_N_3_O_5_	310.1397	0.0	147.0764; 137.0757; 130.0499; 101.0709; 84.0443
Tyr-Arg	5.3	C_15_H_23_N_5_O_4_	338.1821	−0.5	338.1817; 321.1553; 175.1190; 158.0926; 136.0759; 116.0706; 112.0868; 70.0651
Xle-His	5.4	C_12_H_20_N_4_O_3_	269.1607	−0.5	132.1016; 110.0712; 86.0963
Val-Tyr	6.3	C_14_H_20_N_2_O_4_	281.1495	−0.5	182.0813; 165.0548; 136.0759; 72.0808
Tyr-Val	6.5	C_14_H_20_N_2_O_4_	281.1495	−0.3	136.0757; 118.0862; 72.0807
Tyr-Pro	6.9	C_14_H_18_N_2_O_4_	279.1339	−0.3	136.0756; 116.0705; 70.0651
Tyr-Tyr	7.6	C_18_H_20_N_2_O_5_	345.1445	0.0	182.0813; 165.0548; 136.0759
Arg-Phe	7.9	C_15_H_23_N_5_O_3_	322.1873	−0.1	322.1870; 305.1603; 175.1189; 166.0864; 120.0807; 112.0869; 70.0651
Tyr-Xle	9.5	C_15_H_22_N_2_O_4_	295.1651	−0.4	136.0757; 132.1019; 86.0963
Xle-Trp	12.8	C_17_H_23_N_3_O_3_	318.1811	−0.3	205.0971; 188.0705; 159.0917; 146.0600; 86.0964
Phe-Phe	13.2	C_18_H_20_N_2_O_3_	313.1545	−0.6	166.0863; 120.0807
Tripeptides					
Arg-Tyr-Gln	4.6	C_20_H_31_N_7_O_6_	466.2409	0.2	449.2143; 303.1452; 292.1768; 275.1503; 157.1084; 147.0763; 136.0757; 130.0499; 112.0868; 70.0651
His-Xle-Gln	4.9	C_17_H_28_N_6_O_5_	397.2194	−0.1	251.1503; 223.1552; 147.0763; 138.0662; 130.0499; 110.0713; 86.0964
Tyr-Pro-Glu	7.1	C_19_H_25_N_3_O_7_	408.1764	−0.4	245.1130; 148.0601; 136.0756; 70.0651
Arg-Glu-Xle	7.7	C_17_H_32_N_6_O_6_	417.2455	−0.3	304.1613; 287.1348; 269.1242; 258.1558; 243.1338; 241.1293; 157.1084; 132.1019; 112.0869; 102.0549; 86.0964; 84.0444; 70.0652
Thr-Val-Tyr	7.9	C_18_H_27_N_3_O_6_	382.1972	−0.1	201.1234; 173.1285; 155.1179; 136.0757; 101.0709; 74.0600; 72.0808
Val-Arg-Tyr	8.4	C_20_H_32_N_6_O_5_	437.2506	−0.2	321.1556; 274.1873; 239.1502; 175.1190; 136.0757; 112.0869;72.0808; 70.0651
Xle-Xle-Arg	8.5	C_18_H_36_N_6_O_4_	401.2870	−0.2	271.1862; 199.1805; 175.1190; 158.0926; 116.0705; 86.0964; 70.0651
His-Ala-Phe	8.7	C_18_H_23_N_5_O_4_	374.1822	−0.2	209.1033; 181.1084; 166.0863; 120.0808; 110.0713
Phe-Gly-Lys	9.5	C_17_H_26_N_4_O_4_	351.2025	−0.5	205.0971; 204.1343; 187.1077; 177.1022; 147.1128; 130.0863; 120.0808; 101.1073; 84.0808
Xle-Lys-Tyr	9.5	C_21_H_34_N_4_O_5_	423.2600	−0.5	310.1761; 182.0812; 165.0545; 131.1179; 101.1073; 86.0964; 84.0808
Xle-Gly-Tyr	9.7	C_17_H_25_N_3_O_5_	352.1866	−0.3	239.1022; 182.0812; 171.1126; 165.0547; 143.1178; 136.0757; 86.0963
Xle-Gln-Tyr	9.8	C_20_H_30_N_4_O_6_	423.2238	−0.1	225.1233; 208.0968; 182.0812; 165.0546; 136.0757; 131.1179; 101.0709; 86.0964; 84.0443
Xle-Ala-Tyr	10.0	C_18_H_27_N_3_O_5_	366.2022	−0.4	253.1187; 185.1285; 182.0812; 165.0546; 157.1335; 136.0757; 86.0963
Arg-Asn-Xle	10.0	C_16_H_31_N_7_O_5_	402.2458	−0.4	289.1617; 272.1351; 246.1450; 229.1186; 226.1297; 157.1084; 140.0819; 112.0869; 86.0963; 70.0651
Xle-Glu-Tyr	10.2	C_20_H_29_N_3_O_7_	424.2078	−0.1	293.1132; 215.1390; 182.0812; 136.0758; 102.0550; 86.0964; 84.0444
Tyr-Val-Tyr	10.2	C_23_H_29_N_3_O_6_	444.2128	−0.2	235.1443; 182.0812; 165.0545; 136.0757; 72.0808
Val-Xle-Arg	10.5	C_17_H_34_N_6_O_4_	387.2712	−0.5	288.2030; 271.1765; 185.1651; 175.1189; 158.0924; 116.0707; 112.0870; 86.0964; 72.0808; 70.0652
Xle-Pro-Tyr	10.5	C_20_H_29_N_3_O_5_	392.2179	−0.3	279.1337; 183.0493; 182.0814; 165.0545; 136.0758; 86.0963; 70.0651
Glu-Val-Phe	11.1	C_19_H_27_N_3_O_6_	394.1971	−0.4	265.1547; 229.1183; 201.1234; 183.1128; 166.0863; 120.0808; 102.0550; 84.0444; 72.0808
Tyr-Ser-Xle	11.2	C_18_H_27_N_3_O_6_	382.1971	−0.5	251.1026; 233.0921; 223.1077; 219.1339; 201.1234; 136.0757; 132.1019; 86.0964; 60.0445
Xle-Xle-Val	12.0	C_17_H_33_N_3_O_4_	344.2543	−0.2	231.1702; 199.1805; 118.0862; 86.0964; 72.0808
Arg-Tyr-Xle	12.1	C_21_H_34_N_6_O_5_	451.2661	−0.5	338.1821; 321.1556; 295.1651; 292.1762; 275.1500; 175.1189; 157.1084; 140.0818; 136.0757; 112.0869; 86.0964
Xle-Arg-Xle	12.5	C_18_H_36_N_6_O_4_	401.2869	−0.5	288.2028; 271.1760; 253.1658; 175.1189; 131.1178; 112.0869; 86.0964
Xle-Xle-Tyr	12.7	C_21_H_33_N_3_O_5_	408.2491	−0.4	199.1803; 182.0813; 165.0545; 136.0757; 86.0964
Xle-Ser-Phe	13.0	C_18_H_27_N_3_O_5_	366.2023	−0.2	253.1187; 235.1074; 173.1285; 166.0862; 155.1180; 120.0807; 86.0964; 60.0446
Xle-Arg-Phe	13.1	C_21_H_34_N_6_O_4_	435.2713	−0.4	322.1870; 305.1604; 288.2026; 253.1659; 175.1188; 166.0863; 131.1179; 120.0808; 86.0964; 70.0651
Xle-Ala-Phe	13.1	C_18_H_27_N_3_O_4_	350.2072	−0.6	237.1231; 185.1283; 166.0862; 157.1335; 120.0807; 86.0964
Xle-Ser-Phe	13.4	C_18_H_27_N_3_O_5_	366.2022	−0.5	253.1187; 235.1074; 173.1285; 166.0862; 155.1180; 120.0807; 86.0964; 60.0446
Tyr-Xle-Xle	14.5	C_21_H_33_N_3_O_5_	408.2492	−0.2	249.1596; 245.1858; 136.0757; 132.1021; 86.0964
Pro-Xle-Trp	15.1	C_22_H_30_N_4_O_4_	415.2338	−0.3	205.0972; 188.0705; 183.1492; 159.0917; 86.0964; 70.0651
Phe-Arg-Phe	16.9	C_24_H_32_N_6_O_4_	469.2556	−0.3	452.2290; 322.1873; 305.1606; 276.1816; 259.1552; 166.0863; 120.0808; 112.0869; 70.0652
Tetrapeptides					
Glu-Gln-Xle-Gln	6.1	C_21_H_36_N_6_O_9_	517.2617	0.1	258.1084; 243.1339; 230.1135; 147.0764; 129.0659; 102.0550; 86.0964
Glu-Val-Val-Arg	6.6	C_21_H_39_N_7_O_7_	502.2983	−0.1	502.2984; 373.2558; 257.1602; 229.1179; 201.1233; 199.1440; 183.1126; 175.1189; 158.0923; 116.0704; 84.0444; 72.0807; 70.0651
His-Ala-Phe-Glu	8.1	C_23_H_30_N_6_O_7_	503.2249	0.0	209.1033; 181.1084; 148.0608; 120.0808; 110.0713
Lys-Thr-Val-Tyr	8.3	C_24_H_39_N_5_O_7_	510.2922	−0.1	281.1496; 230.1499; 212.1394; 182.0812; 173.1285; 129.1022; 101.1073; 84.0808; 74.0600; 72.0808; 56.0495
Xle-Arg-Xle-Lys	10.0	C_24_H_48_N_8_O_5_	529.3820	0.0	529.3820; 416.2980; 270.1925; 260.1969; 253.1659; 225.1710; 147.1128; 86.0964; 84.0808
Tyr-Gly-Xle-Glu	10.0	C_22_H_32_N_4_O_8_	481.2293	0.0	261.1439; 221.0915; 193.0971; 148.0604; 136.0756; 102.0548; 86.0963; 84.0443
Xle-Arg-Xle-Asn	10.0	C_22_H_42_N_8_O_6_	515.3299	−0.2	515.3300; 402.2459; 385.2194; 270.1925; 253.1659; 229.1183; 131.1179; 112.0869; 87.0553; 86.0964
Thr-Xle-Lys-Tyr	10.1	C_25_H_41_N_5_O_7_	524.3078	−0.2	310.1761; 293.1496; 242.1863; 187.1441; 182.0812; 169.1335; 165.0550; 136.0757; 101.0709; 86.0964; 84.0808; 74.0600; 56.0495
Glu-Val-Phe-Gly	10.2	C_21_H_30_N_4_O_7_	451.2187	0.0	229.1183; 223.1076; 219.1492; 201.1234; 183.1128; 120.0808; 102.0443; 84.0443; 72.0808
Xle-Val-Xle-Arg	10.5	C_23_H_45_N_7_O_5_	500.3555	−0.1	387.2722; 271.1763; 213.1597; 185.1648; 175.1190; 158.0925; 86.0964; 72.0807
Pro-Gln-Xle-Tyr	10.9	C_25_H_37_N_5_O_7_	520.2767	0.2	226.1186; 198.1237; 182.0812; 136.0757; 115.0866; 101.0709; 86.0964; 84.0444; 70.0651
Xle-Ala-Xle-Asp	11.0	C_19_H_34_N_4_O_7_	431.2499	−0.3	247.1291; 199.1808; 185.1284; 157.1335; 86.0964
Gln-Tyr-Xle-Tyr	11.7	C_29_H_39_N_5_O_8_	586.2869	−0.3	292.1292; 247.1077; 182.0812; 165.0545; 136.0757; 101.0709; 86.0964
Pro-Tyr-Val-Arg	11.8	C_25_H_39_N_7_O_6_	534.3035	0.1	274.1870; 261.1234; 257.1608; 235.1441; 233.1285; 175.1190; 136.0757;72.0808; 70.0651
Glu-Val-Phe-Ala	12.0	C_22_H_32_N_4_O_7_	465.2343	−0.1	237.1234; 229.1182; 201.1234; 183.1128; 120.0808; 102.0549; 84.0444; 72.0808
Phe-Arg-Gln-Phe	12.0	C_29_H_40_N_8_O_6_	597.3143	−0.2	450.2459; 287.1503; 240.1455; 166.0863; 120.0808; 101.0709; 84.0444
Val-Xle-Pro-Tyr	12.1	C_25_H_38_N_4_O_6_	491.2865	0.2	279.1339; 185.1648; 182.0913; 86.0964; 72.0808; 70.0651
Val-Arg-Tyr-Xle	12.1	C_26_H_43_N_7_O_6_	550.3346	−0.3	550.3348; 434.2398; 402.2136; 391.2452; 303.1452; 295.1652; 256.1768; 239.1503; 211.1553; 136.0757; 132.1019; 117.1022; 112.0869; 86.0964; 72.0808
Xle-Thr-Glu-Phe	12.3	C_24_H_36_N_4_O_8_	509.2606	0.0	277.1183; 213.0870; 203.1026; 197.1285; 185.0921; 166.0863; 120.0808; 102.0550; 86.0964; 84.0444: 74.0600; 56.0495
Glu-Asn-Xle-Xle	12.6	C_21_H_37_N_5_O_8_	488.2714	−0.1	244.0925; 227.0663; 226.0821; 199.0712; 132.1018; 129.0659; 102.0549; 86.0964; 84.0444
Trp-Xle-Gln-Pro	13.0	C_27_H_38_N_6_O_6_	543.2926	0.0	272.1757; 255.1490; 226.1184; 187.0865; 159.0916; 116.0706; 101.0709; 86.0964; 84.0444; 70.0651
Tyr-Pro-Glu-Xle	13.0	C_25_H_36_N_4_O_8_	521.2605	−0.1	358.1973; 243.1339; 227.1026; 199.1077; 136.0757; 102.0550; 86.0964; 84.0444; 70.0651
Ala-Xle-Xle-Glu	13.1	C_20_H_36_N_4_O_7_	445.2656	−0.1	199.1801; 185.1286; 157.1335; 148.0602; 102.0550; 86.0964
His-Xle-Ser-Phe	13.3	C_24_H_34_N_6_O_6_	503.2611	−0.3	251.1501; 223.1552; 110.073; 86.0964
Glu-Pro-Phe-Val	13.4	C_24_H_34_N_4_O_7_	491.2500	0.0	245.1287; 227.1025; 217.1335; 199.1076; 120.0807; 102.0550; 84.0443; 70.0651
Tyr-Pro-Xle-Val	13.6	C_25_H_38_N_4_O_6_	491.2864	−0.1	261.1231; 233.1282; 211.1439; 183.1491; 136.0756; 118.0963; 86.0963; 70.0651
Tyr-Gly-Xle-Xle	14.0	C_23_H_36_N_4_O_6_	465.2707	−0.1	193.0972; 171.1128; 136.0757; 86.0964
Xle-Ala-Tyr-Phe	15.1	C_27_H_36_N_4_O_6_	513.2708	0.0	207.1127; 157.1335; 136.0757; 86.0964
Xle-Xle-Arg-Phe	15.4	C_27_H_45_N_7_O_5_	548.3553	−0.3	548.3555; 435.2714; 366.2500; 355.2816; 322.1874; 305.1608; 253.1659; 227.1754; 199.1805; 166.0863; 120.0808; 112.0869; 86.0964
Pro-Ser-Phe-Phe	15.5	C_26_H_32_N_4_O_6_	497.2393	−0.4	207.1126; 167.0815; 166.0865; 157.0972; 120.0807; 70.0651
Xle-Ser-Lys-Phe	15.5	C_24_H_39_N_5_O_6_	494.2971	−0.4	329.2175; 294.1809; 216.1343; 201.1234; 198.1238; 173.1284; 170.1288; 166.0862; 155.1179; 129.1022; 120.0808; 101.1073; 86.0964; 84.0807; 60.0444
Xle-Asp-Xle-Xle	15.9	C_22_H_40_N_4_O_7_	473.2970	0.0	229.1179; 201.1233; 183.1129; 86.0964
Xle-Arg-Phe-Phe	16.9	C_30_H_43_N_7_O_5_	582.3396	−0.4	452.2291; 400.2356; 389.2660; 270.1925; 259.1553; 253.1659; 166.0963; 131.1179; 120.0808; 112.0869; 86.0964
Xle-Pro-Met-Trp	17.2	C_27_H_39_N_5_O_5_S	546.2746	0.2	342.1846; 229.1005; 211.1441; 205.0972; 188.0706; 183.1492; 159.0917; 104.0528; 86.0964; 70.0651
Xle-Pro-Phe-Xle	17.5	C_26_H_40_N_4_O_5_	489.3071	−0.1	245.1280; 217.1333; 211.1443; 183.1491; 120.0809; 86.0964; 70.0652

## Data Availability

Not applicable.

## Supplementary Materials

The following are available online, Table S1: Retention times (Rt, min), proposed formulas, adducts, molecular weights, experimental *m/z*, accuracy (Δ, ppm), main diagnostic product ions, and GRAVY values of the 79 tentatively identified short endogenous peptides in whole milk, whole whey, skimmed whey, and permeate whey. Table S2: Subdivision of the annotated short peptides according to the four subgroups (SP1, SP2, SP3, and SP4). Report of the manual search of the annotated peptides in caseins and whey protein sequences and report of the bioactivity search of the annotated peptides on Milk Bioactive Peptide Database (MBPDB), BIOPEP, and Peptide Ranker.

## Sample Availability

Samples of the compounds are not available from the authors.
